# Integrating climate change adaptation policies in spatial development planning in hyperarid regions of Kerman province, Iran

**DOI:** 10.1016/j.heliyon.2023.e19785

**Published:** 2023-09-02

**Authors:** Hossein Karami, Romina Sayahnia, Shahindokht Barghjelveh

**Affiliations:** Department of Environmental Planning and Design, Environmental Sciences Research Institute, Shahid Beheshti University, Tehran, 1983969411, Iran

**Keywords:** Climate change scenarios, Human development, Land cover, Supply and demand

## Abstract

In recent years, lifestyle changes and urbanization of societies, as well as macro-environmental changes, i.e. climate changes (CCs), have caused changes in the land spatial structure and the transfer of resources between different economic sectors of the land. The development of long-term spatial development plans (SDPs) needs to be compatible with CCs, especially in hyperarid areas with low supplies and high demands. In this research, machine learning methods; including Cellular Automata (CA), Random Forest (RF) and regression models through PLUS model were used to simulate the amount of supplies and demands based on land cover (LC) maps during the years 2000, 2010 and 2020 in the hyperarid areas of Kerman, Iran. Then, the best predicted model (Kappa = 0.94, overall accuracy = 0.98) was used to simulate changes in LC classes under climate change scenarios (CCSs) for 2050. The results showed the efficiency of machine learning in simulating land cover changes (LCCs) under CCSs. Findings revealed that SDPs of these areas are not compatible under any possible consideration of CCSs. The modeling results showed that spatial development plans under CCSs is not environmentally efficient and there is no compatibility between supplies, based on agricultural lands, and demands, based on increased population, by 2050. Overall, under the scenario of RCP 8.5, man-made, agriculture and natural LC classes with 106.9, 2.9, and 18.6% changes, respectively, showed the greatest changes compared to 2020. Population control, adjustment of infrastructures, and changes in LC plans can reduce socio-economical and socio-environmental problems in the future of hyperarid areas to some extent.

## 1 Introduction

Human development depends directly on the land systems which have changed substantially over the last century [1,2]. On one hand, human development and spatial development plans (SDPs) can be severely influenced by climate change (CC), and adaptation strategies should be considered in the future planning [1,3]. On the other hand, the spatial imbalance during rapid economic growth (population increase) is a very big obstacle to sustainable spatial development (SSD), which includes the mismatch between the environment and the economic and demographic structure, and as a result, excess demand and excess supply will somehow cause imbalance [4,5].

Overall, CC refers to changes in the climate that occur over several decades or more [6,7], in which analyzes of the latest climate observations show that the average temperature of the earth's surface in recent decades, is 1.1 °C higher than the pre-industrial (1850–1900) [8]. Also, according to NASA's estimates, from 1880 to 2020, the average temperature of the earth has warmed by 1.18 ᵒC, and the highest temperature in the 140 years recorded was in 2017 [9]. The CC is beyond the local scales and has global effects. So that in the long term, the negative effects of CC are much more than its positive effects, and dry and hot (hyperarid) countries are more affected by these changes [5,10,11].

Due to the difference in geographical, climatic conditions and economic/social structures, CC has different effects around the world. It has very significant effects on human well-being, either directly, such as food production/water crisis, or indirectly, such as changes in the geographic distribution of animal/plant populations [12,13], and change or limitation of habitats [14].

Food security threat is one of the most important effects of CC through the reduction of arable land for agriculture, which coincides with the increase in population [1,14]. Moreover, at the same time as industrial development, there is a significant relationship between the percentage of urbanization and the development of countries (i.e. the United States with 82% and Japan with 91% of urbanization), so that it is predicted that more than 70% of the world's population will be urban by 2050 [15]. Therefore, the growth of urbanization means the transfer of resources from the agricultural sector to cities, which has caused many environmental and socio-economic problems, i.e. pollution, lack of resources, and land degradation and conversion [2,16].

Land cover (LC) and its changes is one of the most important factors in spatial development plans (SDPs) that will be strongly affected by the CCs. The LC plays a very important role in determining the carrying capacity/supply of each region [4,17]. And modeling of land cover changes (LCCs) under CC plays an important role in population distribution and development of transportation infrastructure planning for the future [[18], [19], [20]].

Moreover, the SSD is achieved as a result of the balance between economic, environmental and social benefits during the SDPs, which is the spatial matching of supply and demand for the development, i.e. economy, society and environment, and surplus demand and excess supply will both cause imbalance in some way [4]. In this way, SDPs should have the ability and flexibility, and systematically have solutions to face the effects of CC and to have appropriate reactions against various threats [2].

There is a close relationship between CCs and LCCs, and studying and investigating the relationship between them through mathematical and statistical models is an important part of environmental planning [21]. And appropriate SDP can somewhat reduce the negative effects of CC on societies [7]. However, using an adaptation approach, is one of the characteristics of the strategic plans and on the other hand, one of the requirements of efficient SDPs [22]. Adaptation is one of the characteristics in SP, especially, where CCs and changes in economic and social policies or technological changes may lead to the selection of options substitutions and change of procedure or in other words adaptability [23,24]. Therefore, it is necessary to evaluate the LCCs for suitable SSP using accurate and cost-effective methods.

Yet, various studies of modeling and predicting LCCs have been conducted around the world [19,25,26] in order to evaluate the LCCs and better manage land for the future [27]. The modeling tries to predict future LCCs based on past trends and factors driving change. One of the advantages of different LCCs simulation models is the reproducibility and scenario of driving factors, including human plans and external environmental factors such as CC [28]. Moreover, CC must be carefully simulated and evaluated due to its very effective role in changing land use patterns [29]. Therefore, by knowing the LCCs over time, it is possible to predict future changes and take the necessary measures.

Among the methods used, Cellular Automata (CA) [30], Markov Chain Model (MCM) [31], Artificial Neural Network (ANN) [32], Automated Cellular Fusion Methods with Markov Model (ACFM-MM) [33], Scenario Generator (SG) [34], SLUCE [35], GEOMOD [36] are the best known models for the LCCs evaluation.

In this regard, CA is one of the best methods for simulating LCCs over time, but it may have limitations that combining it with other models such as MCM will lead to better results [37]. Also, the combination of Patch-Generating Land Use Simulation (PLUS) with CA has a high ability for modeling LCCs [38]. However, there is no detail information about the adaptability of LCCs during the SDP under CC, especially in hyprarid lands.

In the central regions of Iran with a dry climate and average rainfall between 50 and 100 mm, SD is very vulnerable to CC [39]. According to the evidence, a dry weather regime with a decrease in precipitation will prevail in the dry regions of Iran, especially in the hot seasons [40]. In such a way that Iran will face an average temperature increase of 2.6 ᵒC and a decrease in annual precipitation of about 35% in the coming decades [41].

On the other hand, CC in Iran will lead to a significant decrease in agricultural products, which will lead to negative economic and social effects due to the dependence of many people on agriculture. Also, health issues and mortality caused by drought will increase, especially in densely populated cities, and food security will be threatened due to reduced production and pressure on limited water resources [41]. In this regard, Kerman province, due to its size and considerable population, located in central Iran, has been severely affected by CC.

As a result of these environmental changes that have negative or positive effects on the SD, they will influence the achievement of the land planning goals and prospects and should be taken into consideration in the strategic development and adaptation mechanism in spatial development plans (SDPs). And due to the fact that Kerman is located in a hyprarid arid area with severe climatic effects and also the need to prepare a spatial development document and the future development of the agricultural sector, this area was selected for study. Therefore, this research evaluates the adaptability of SDPs based on the climate change scenarios (CCSs) [Representative Concentration Pathway; RCP2.6, RCP4.5, RCP6, RCP8.5] during the recent and future decades through simulation in the central hyperarid regions of Iran. The second objective is to study the adaptability between supply (LC) and demand in the concept of SDPs in the form of balance or imbalance of its components under different CCSs in Kerman province.

## 2 Materials and methods

### 2.1 Study area

**Fig. 1:**
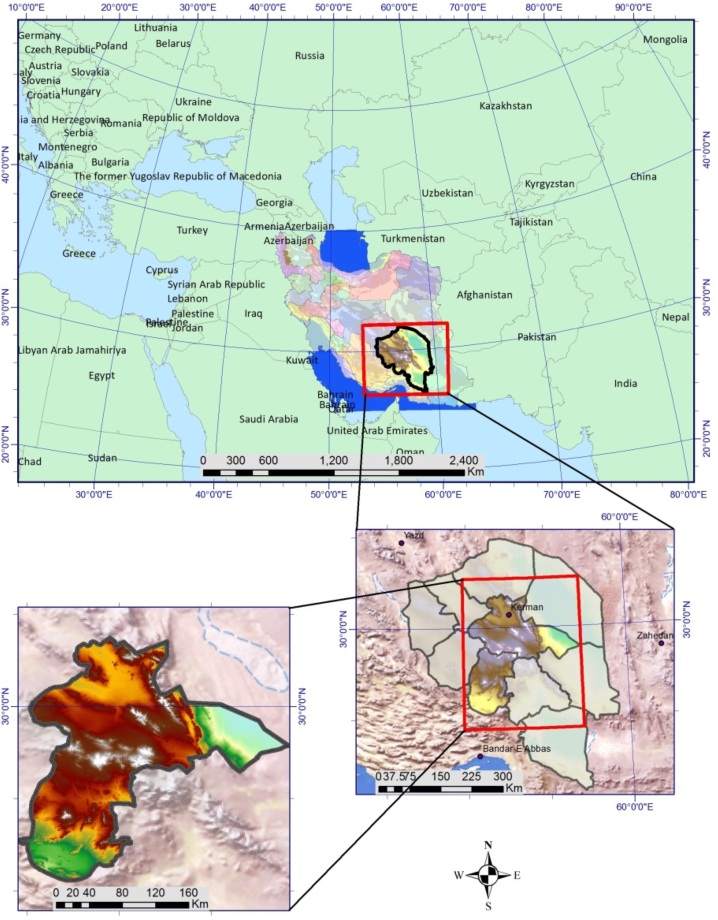
Geographical location of the study area in Iran.

Kerman province is considered as the largest province of Iran with 15.11% of the total area of the country, is located in the southeastern part of Iran. Kerman located at an elevation of 1760.0 m above sea level. In this region, the summers are hot and arid. Yearly temperature is 16.48 °C. Kerman typically receives about 23.9 mm of precipitation annually. Due to its special geographical location and existence of rich mineral resources, this province has a very important role in transit and transportation. Kerman-Bardsir and Baft-Rabor-Borzovieh planning regions (4635388 ha), were selected as a case study in this research and the model was designed using the data of these regions have been implemented (Fig. 1).

### 2.2 Research methodology

The evaluation of the SDP used the concept of matching supply and demand from the perspective of required use (demand) and possible use (supply). For this purpose, using the information and the data extracted from the SDPs and CCSs, the LC maps are simulated for the horizon 2050.

### 2.3 Simulation

Agent-based models, artificial neural networks (ANNs), cellular automaton models (CA), Markov chain(MC), random forest, regression models and other optimization models are among the most widely used models [42]. In this research, machine learning algorithms implemented in the form of PLUS model were used. Machine learning algorithms are able to solve complex problems through different inputs, and since LC data has a nominal and ordinal scale, these methods are non-parametric and more accurate than traditional parametric methods, that are more appropriate [25]. In recent years, the use of machine learning methods in order to model LC and improve them, has made it possible to model the future LCCs [17].

### 2.4 Driving forces of land cover changes (LCCs)

In this research, five layers of data were used as driving forces for LCCs as described in Table 1.

**Table 1:** Information layers and driving forces of land cover changes (LCCs).

Description	Source	Driving force (information layer)	Scale^a^
The ten-year average temperature and precipitation were calculated in the three decades of 1990, 2000, and 2010, and four climate scenarios were used for the horizon calculations.	Meteorological Organization data and statistical calculations	Average rainfall/Average temperature	100 m - Yearly
The weighted distance from the roads was calculated according to freeway, highway and main road as well as railways.	Data from the Ministry of Roads and Urban Development Spatial information database of the national land survey document	Distance from the road	1:25000
The population density at the district level was calculated using census data for three decades as well as population estimates in Kerman Province study.	Population and housing census of 1996–2006 and 2016 Research studies of Kerman province	Population density	1:25000
–	SRTM	Digital elevation model (DEM)	30 m
Similar classes were merged and classified into 6 main land cover (LC) classes.	Land cove (LC) European Space Agency	Land cover (LC)	300 m

a All data converted to the 100-m resolution raster format.

The Intergovernmental Panel on Climate Change (IPCC) has used four scenarios based on the amount of radiative forcing of the atmosphere-Earth system in 2100 to represent the concentration course (RCP) of greenhouse gases, especially carbon dioxide; Accordingly, four scenarios named RCP2.6, RCP4.5, RCP6 and RCP8.5 have been designed [6]. The climatic variables used in this research have been used using the CCSs mentioned in the fifth report (IPCC) for the horizon 2050 which has been simulated by the National Meteorological Organization for different decades (Table 2).

**Table 2:** IPCC scenarios used for the simulating cover change in horizon 2050 in the study area.

Climatic variables Scenario	Precipitation (%)	Temperature (ᵒC)
RCP 2.6	+10	+0. 7
RCP 4.5	−20	+1.25
RCP 6.0	+10	+1.5
RCP 8.5	−20	+2.5

Using data from 2000 to 2020, the average rainfall and temperature maps under the 4 CCSs corresponding to 2050 were calculated using ArcGIS software. In order to model and use the time series data of the spatial structure of the study area, maps of transportation infrastructures including freeways, highways, main roads and railway lines of this area in the three decades of 2000, 2010, 2020 and also the horizon of 2050 were prepared based on the spatial access. Also, based on the census data of the last three decades and based on the estimates of Kerman province's research document, population density maps related to these four time periods were prepared. The LC map related to the end of the three decades of 2000, 2010 and 2020 was prepared and based on the proximity of the LC classes; it was converted into six main classes (Results are not presented here).

### 2.5 Simulating the land cover map for the future

**Fig. 2:**
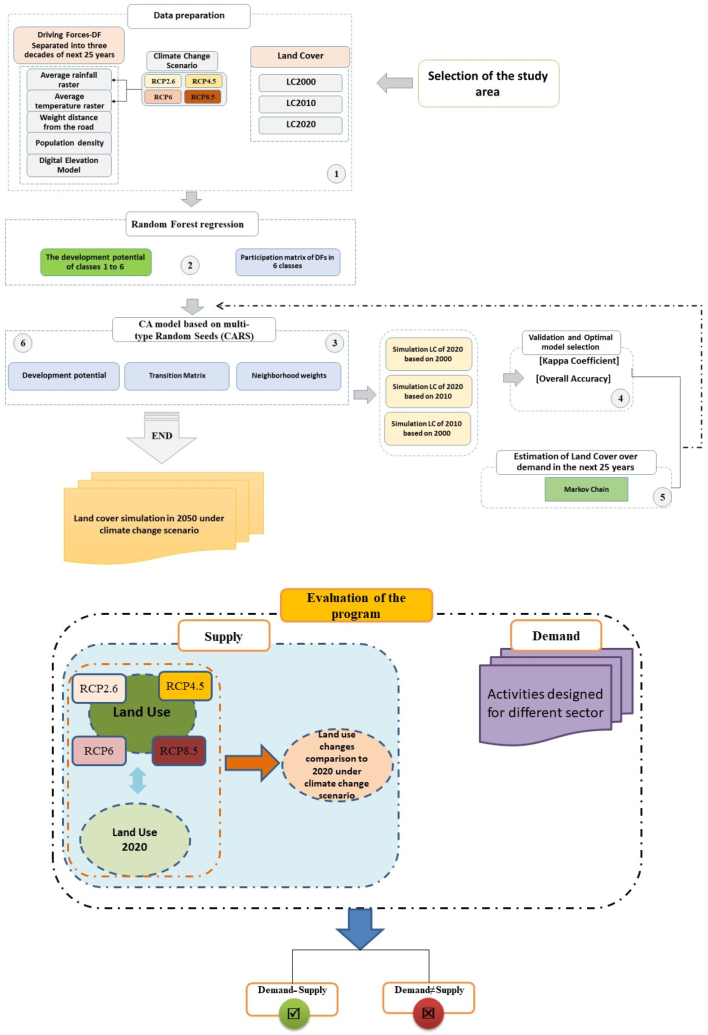
Schematic view of the land cover (LC) simulation process (top) and evaluation process of the study program from the perspective of adaptation to CC (below) for the year 2050 in study area.

Considering that the scale of LC in this study is considered to be a province, the effects of changes in macro climatic variables on LC were investigated and modeled. In order to predict and simulate the LC map in the future 25-year time horizon, the PLUS model was used. Among the models developed for simulating land use (i.e. Logistic-CA, ANA-CA, CLUE_S, CLUMondo, FLUS), the PLUS model is more accurate for simulating the distribution of LC classes, especially natural covers, and simulates the future use using the Random Forest algorithm [17]. The LC modeling using the PLUS model was carried out as follows (Fig. 2: top).

First, the land uses related to the beginning and end of the three decades of 2000, 2010 and 2020 were prepared and due to the dry and expressive climate of the study area and as a result of the small area and the proximity of some land cover classes, 26 primary classes were merged and six The major class was obtained. In order to be able to choose from a pair of users who are the best option for simulating the future user (2050), we must simulate the existing users two by two. Using three years of LC, three models were simulated. Moreover, to choose the appropriate model for simulating the LC in the future, three decades of LC were used and the optimal model was selected based on the lowest error coefficient. For this purpose, using the LEAS module, the development potential of each of the LC classes was prepared.

Random Forest Regression (RFR) algorithm was used to extract these layers in order to learn and sample classes. The parameters used in this research for RFR are as follows:

Number of Regression Tree: 20.

Sample Rate: 0.01.

Mtry: 5.

Using the RFR model and driving force layers, the effectiveness of LCCs, or in other words, the contribution of each of the mentioned forces in shaping and determining each of the LC classes, was also calculated.

Using the Cellular Automata model based on Random Forest (Called CARS in PLUS Model), and data of the years 2010 and 2000 and driving forces, the LCC of the years 2020 and 2010 was simulated (3 modes in total). In order to evaluate and select the optimal land use prediction model, Kappa coefficient and Overall accuracy were used. The closer the kappa coefficient and overall accuracy are to 1, the higher the accuracy of the model for simulating future uses. Then, for four different RCP scenarios, maps of driving forces of temperature and precipitation for 2050 were prepared using ArcGIS software tools. To estimate the demand of each LC class, the Markov Chain method was used in the PLUS model, and the Transition Matrix was prepared for the target year. Then, based on the optimal model that had the least error, 4 LC maps were simulated using the CARS model in these scenarios for the year 2050.

The method of measuring the adaptation of the training program to CCs was done through the adaptation of possible changes to the corresponding plans of each sector (Fig. 2: below). In fact, the compatibility evaluation of a plan was done through overlapping between the layer of LCCs and related sector.

## 3 Results

**Fig. 3:**
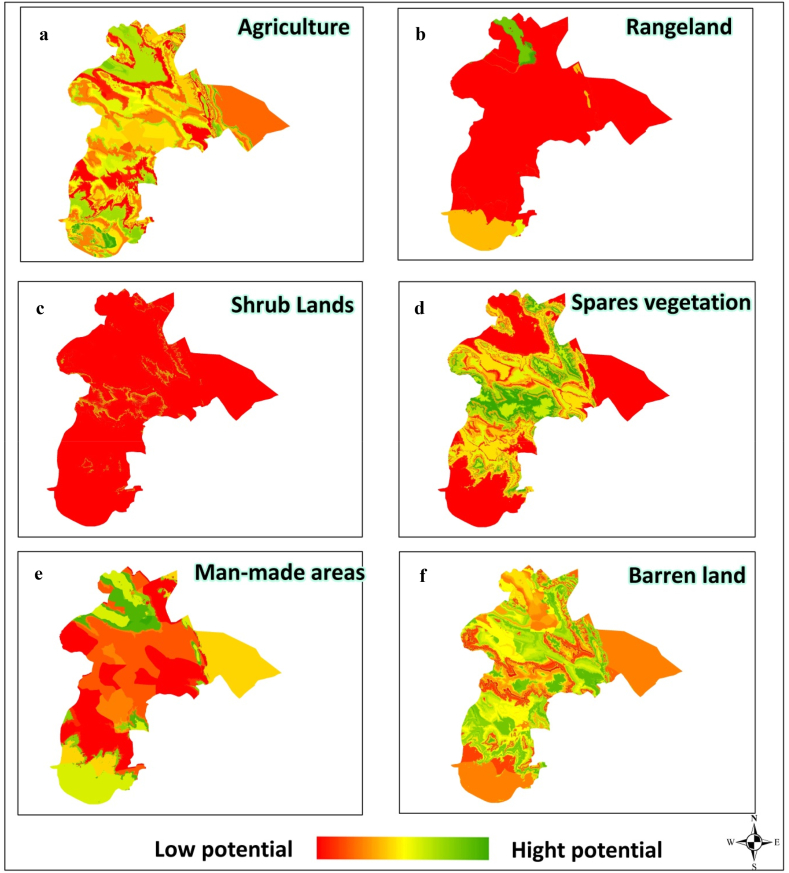
Development potential of land cover (LC) classes in agriculture (a), rangeland (b), shrub land (c), sparse vegetation (d), man-made areas (e) and barren land (f) uses.

The figure below shows the simulation results of the development potential of each of the LC classes based on the lowest error coefficient obtained using the LEAS method (data not presented). In general, according to the climatic nature, temperature and humidity characteristics of the region, the shrub cover has the highest potential in this region. Shrubs have often poor condition compared to other vegetation in terms of annual growth and food production (Fig. 3(. In 2020, 0.42% of the area has been under human construction. Also, a large part of the region (82%) has a high potential for barren lands. And only 8.5% have high potential for production (agriculture).

As driving forces, the LC layers classes were extracted using Random Forest Regression (RFR) algorithm. The parameters used in this research for RFR are as follows:

Number Of Regression Tree:20.

Sample Rate:0.01.

Mtry: 5.

**Fig. 4:**
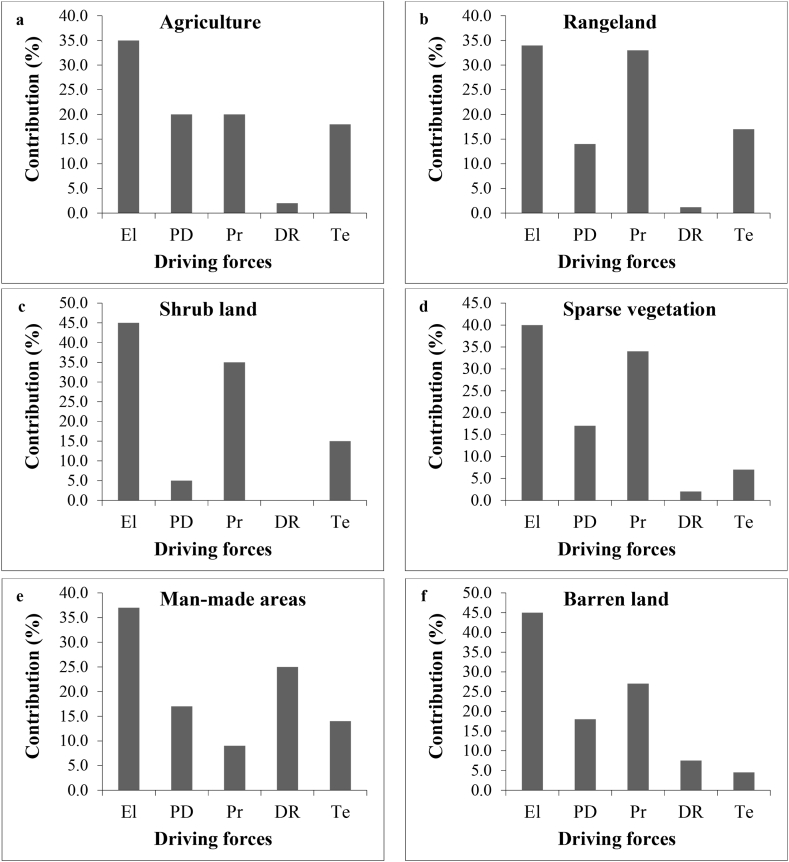
The participation rate of each driving force in agriculture (a), rangeland (b), shrub land (c), sparse vegetation (d), man-made areas (e) and barren land (f) in 2010–2020 (El = Elevation, PD=Population density, Pr= Precipitation, DR = Distance from the road, Te=Temperature).

The effectiveness of LCCs, or in other words, the degree of participation of each of the mentioned forces in shaping and determining each of the LC classes, which has been calculated using the RFR model and driving force layers (Fig. 4). The results of the evaluation of the driving forces in determining the type of land use show that the impact of driving forces is different depending on the type of land use. In general, in agricultural use, elevation has had the greatest impact (35%) (Fig. 4: a). In rangeland use, rainfall and elevation (34 and 33%, respectively) had the greatest impact (Fig. 4: b). Also, for man-made lands, the factors of elevation and distance to the road (32 and 25%, respectively) have been effective (Fig. 4:e). Also, other uses showed similar trend (Fig. 4: c, d, f).

**Fig. 5:**
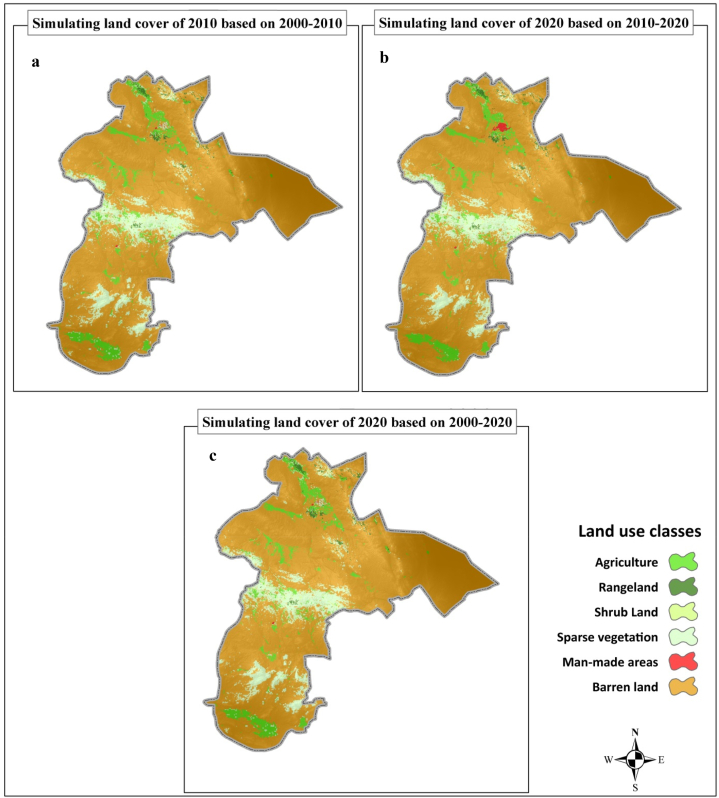
Simulated land cover (LC) map for 2020 using different time series data of 2000–2010 (a), 2010–2020 (b) and 2000–2020 (c).

Based on the results within a LC class, the role of each driving force is different from each other; For example, in the agricultural class, elevation, precipitation, population density, temperature and distance to the road network determine its development or reduction, respectively. Also, the amount of participation (role) of each of the driving factors in the development of each of the LC classes is different compared to each other. For example, the role of rainfall in the development of rangelands is higher than its role in the development of built land. In almost all classes of LC, elevation and precipitation play a more decisive role than other factors, although in the case of built-up lands, the distance to roads plays a more effective role than precipitation. The result of simulating LC maps using the CARS in three different models and using pairs of maps from 2000 to 2010 and 2020. 2000–2010 and 2000–2020 are presented as fallows (Fig. 5). The assessments show that, for example, from 2000 to 2020, man-made lands with the most changes (160% growth) have increased from 7559 ha to 19655 ha. Also, during this period, agricultural lands have reached 271922.5388 ha with a growth of 2.3% from 265785 ha. In the same way, rangelands, barren lands, shrublands, and barren lands have had growth equivalent to −0.19, −2.3 and −2.1%, respectively (Fig. 5).

To compare the accuracy of the models, the Kappa coefficient and the overall accuracy of the output maps of the three models is shown in Table 3. In general, simulated maps from 2010 to 2020 with a factor of 0.940 and 0.981 for kappa and overall accuracy, respectively, has shown higher efficiency compared to the two periods of 2000–2010 and 2000–2020 (Table 3).

**Table 3:** Accuracy evaluation results of simulated models.

Time Series	Kappa Coefficient	Overall Accuracy
2000–2010	0.904	0.971
2010–2020	0.940	0.981
2000–2020	0.861	0.956

**Fig. 6:**
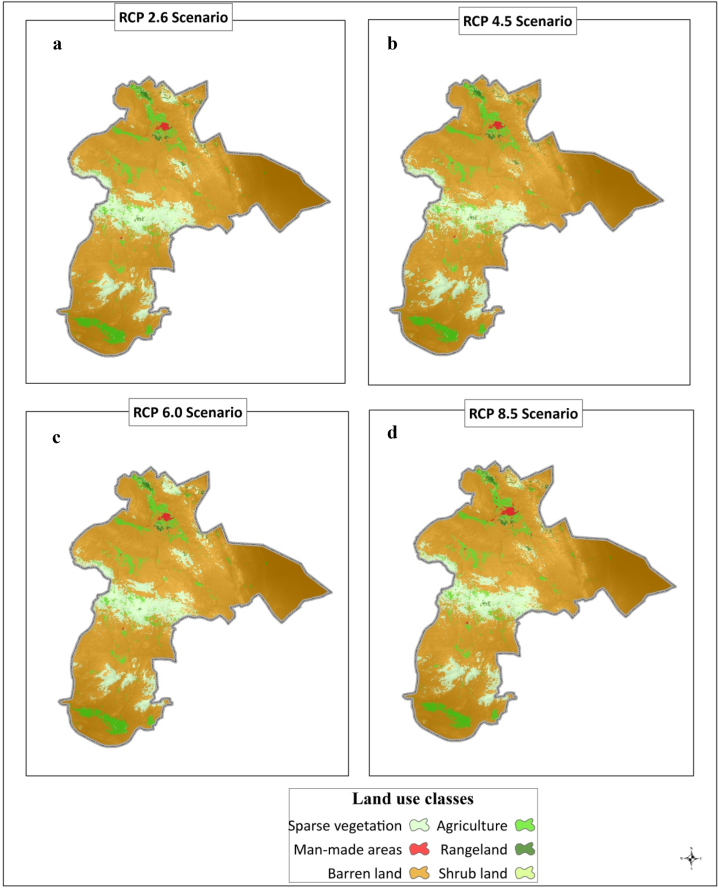
Simulated maps of land cover under climate change scenarios RCP2.6(a), RCP4.5 (b), RCP6.0 (c) and RCP8.5 (d) for 2050.

Considering that the model related to 2010–2020 has the most accuracy in both evaluated factors and was chosen as the selected model for the simulation of LC in 2050. The LC map in 2050 for four CCSs is shown separately as follows (Fig. 6). The results of the evaluation of the changes in land use under different methods showed that each of the land uses will be very intense over time and taking into account the CC. Under scenario RCP 2.6, the amount of changes in agricultural land, rangeland, shrublands, sparse vegetation, man-made lands and barren lands is 2.8, −1.5, −3.6, 18.7, 106.9 and −3.1%, respectively. Under the scenario RCP 4.5, the amount of changes in agricultural land, rangeland, shrublands, sparse vegetation, man-made lands and barren lands is equal to 3.3, −1.4, −2.9, 18.6, 106.8 and −3.1%, respectively. The amount of changes in agricultural land, rangeland, shrublands, sparse vegetation, man-made land and barren land under scenario RCP 6.0 is equivalent to 2.8, −1.5, −3.6, 18.7, 106.9 and −3.1%, respectively. Also, under scenario RCP 8.5, the changes in agricultural lands, rangelands, shrublands, sparse vegetation, man-made land and barren lands are equivalent to 2.9, −1.5, −3.7, 18.6, 106.9, and −3.1%, respectively.

**Fig. 7:**
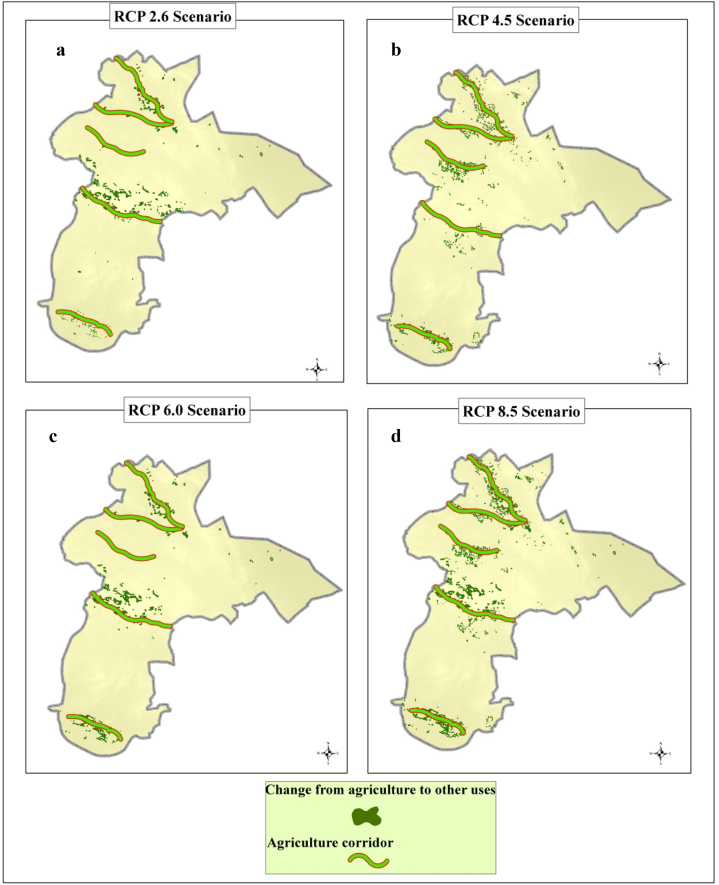
Overlap of LCCS from agriculture cover to other classes with agricultural corridors of 2050 horizon in scenarios RCP2.6(a), RCP4.5 (b), RCP6.0 (c) and RCP8.5 (d).

The results of measuring the adaptation of the land development to CC were carried out through the adaptation of possible changes to the corresponding plans of each sector. As an example, the plans of the agricultural sector are compared with the changes in crop cover to other uses, and in case of compliance, the plan of the agricultural sector is compatible with CCs and in case of non-compliance, it is incompatible. The findings of this research indicate that the change of agriculture cover to other covers under all four CCSs is highly compatible with the agricultural development corridors predicted in the document of Kerman Province (Fig. 7). Based on the CCs in the region, the conversion of natural lands to agriculture or agriculture to man-made or other uses have been different. In general, during scenarios RCP 2.6, RCP 4.5, RCP 6.0 and RCP 8.5, approximately 7687.0, 8941.6, 7687.0 and 8020.4 ha, respectively, of agricultural land will be converted to other uses.

Under all CCSs, there is an obvious mismatch between the changes of LC to other classes and the development of the designed agricultural corridor. This discrepancy is different in different scenarios, and it is observed that in the RCP8.5 scenario, which has more severe effects for CC compared to other scenarios, all the corridors are located in areas that face a decrease in the level of agriculture cover. Under the other three scenarios, the conditions are almost the same, with the difference that the crop cover will not decrease around the middle corridor designed in the region. Therefore, from this point of view, it can be concluded that based on the evidences related to this region in the agricultural sector and in none of the 4 CCSs does not have the necessary compliance and compatibility, and specific policies and strategies should be considered for this inconsistency (Fig. 7).

## 4 Discussion

Taking into account the CCSs in the hyperarid area of Kerman, showed the imbalance in SDPs. In other words, considering the severe CCs until 2050 in these hot and dry regions, the development of the economic and social sector, which will be accompanied by more demand than the agricultural sector, will not be effective. In fact, during the last decades, the frequency of heavy rains in Iran will decrease and the temporal and spatial displacement of rains will occur [43]. In such a way that spatial balance is important in land development under variable environmental conditions [5]. The results of this research showed that the simulation of the effects of CCs on LCCs through machine learning models and algorithms make it possible to evaluate the experimental plans developed on this basis and provides a framework based on which the compatibility of the plans can be measured.

Simulating the effects of CC under all four scenarios indicates that the changes in LC and the consequent change in the supply of the environment will lead to a change in the spatial structure of the region. In this dry and hot region, agriculture as supply severely reduced over time. The most land changes were observed under scenario RCP8.5, which will be affected by the increase in temperature, decrease in rainfall and increase in population. These effects indicate that land-based climate mitigation in developing regions might have severe consequences that are in conflict with the achievement of SSD [1]. Therefore, it will be possible to adapt to environmental changes in order to achieve spatial balance through the accurate prediction of the effects of CCs on the spatial structure of a region and, accordingly, to predict management strategies in spatial plans.

However, many of the effects of CC are unknown, and the extent of its effects has not yet been accurately estimated, and there are many uncertainties in the estimation of these effects. It may even have positive effects in the short term such as less need for heating in winter or in increasing some agricultural production. Therefore, adaptability is one of the basic characteristics of an educational program, and under different CCs, it should have appropriate plans and have the necessary flexibility. In previous study also it was proven that most of China's prefecture-level cities are spatially imbalanced in regional development, with a common phenomenon of overexploitation based on spatial supply capacity and development demand intensity [4]. Similarly, by modeling the LCCs under CCSs up to the year 2100 in Sub-Saharan Africa, it was reported that LC plays a crucial role in ambitious mitigation scenarios where the scenario output can improve interactions between CC and spatial plans [1].

The evaluating and monitoring the SDPs can lead to the identification of the unforeseen effects of destructive environmental changes [14,16]. Overall, the evaluation of cumulative effects resulting from environmental factors implicitly reflects the degree of compatibility of SDPs with external factors. Therefore, the SDP is the dynamics of the environment and increasing uncertainties or different scenarios that has led planners to flexible (or adaptive) planning, and this flexibility can be achieved through monitoring and evaluation mechanisms [14,43].

Territorial policies or SDPs are one of the most important driving forces of LCCs in the future, which will cause changes in the spatial structure and consequently changes in society [22]. On the other hand, macro-trends such as CC will have a very important and destructive effect on various sectors such as food production, water, health, ecosystem and human habitats and infrastructures. This effect is much more intense in developing countries with income levels [44].

The type of LC as an important factor in the spatial structure of each region can be changed under the influence of driving forces include socioeconomic, cultural, political, technological and natural factors [22]. This change in lands leads to a change in the amount of supply in a region and consequently changes the regional balance [5]. Previusly, different models, e.g. Cellular automata Markov (CAM) model was used, for the simulation to predict urban spatial growth based on supply and demand chain and mentioned that adaptation of sustainability in the developing cities requires clear and explicit definitions that are embedded with the recent and particular context [3].

Agricultural products are strongly affected by CC. On one hand, LC also plays a crucial role in ambitious mitigation CCSs [1]. According to the developed models, as a result of CC, agricultural production will decrease by 17%. Also, due to the effect on the amount of supply and as a result of high demand, the price of some products will increase, and as a result, it will be harder for poor people in rural areas to access food [[2], [45]]. Adaptation may be spontaneous and individual like farmers or planned and at the level of governments and decision makers. Also, adaptation may be in the form of tactics and short-term, ranging from daily to one-year time frame, or in the form of long-term adaptation, based on forecasts and taking into account temporal and spatial uncertainties, which in this case, as the strategy is important [45]. In general, demographic changes and increased demand, especially in dry and hot regions, strongly affect environmental sustainability. Therefore, proper SDP is necessary to reduce the effects of CCs and adapt land development to these changes [5,43], in which by providing high resolution data, the scenario output can improve interactions between CC and LCCs [1].

Overall, among the of this research is the lack of access to the LC layer in the past decades (before the 1990s) and digital data related to the driving forces of changes in older time periods. However, in this research, we tried to overcome this problem as much as possible by using the data of three different time periods. Also, another limitation is the need to use continuous raster data, which inevitably the available data that are discrete in nature (such as roads) in this research was transformed into the access to roads and as a continuous data register was used. Moreover, in this research, the data related to precipitation and temperature have been used in the form of continuous data interpolation, which is the use of continuous data from satellite images that have the ability to continuously map the temperature of the earth's surface and the amount of precipitation will cause higher accuracy. Furthermore, in the forecasting models, it has been assumed that there is no political interference and external macro-trends (external driving forces) such as geopolitical factors and technological changes, which seem to be factors influencing the trends of land cover changes [22]. Therefore, in the future research, simulating other factors and especially the effects of technology on human needs (the demand of each type of LC) and supply (the amount of land required for each type of production) will be one of the most important challenges.

## 5 Conclusion

In this research, the SDPs of hyperarid area of Kerman province was evaluated from the perspective of the effects of CCSs up to the horizon of 2050. The modeling results showed that under the CCSs and reduction of rainfall in the next decades in this region, there is no compatibility between the supply based on agricultural land and the demand based on the increase in the population living in this region. In other words, the land irrigation plans that are being implemented are not ecologically and environmentally efficient for the future of this region. Population control, adjustment of the infrastructure and changes in land management procedures can, albeit partially, increases the adaptability of the land plans and to some extent reduce economic-social-environmental problems in this region.

## Author contribution statement

Hossein Karami: Performed the experiments; Analyzed and interpreted the data; Contributed reagents, materials, analysis tools or data; Wrote the paper.

Romina Sayahnia: Conceived and designed the experiments; Analyzed and interpreted the data; Contributed reagents, materials, analysis tools or data; Wrote the paper.

Shahindokht Barghjelveh: Conceived and designed the experiments; Contributed reagents, materials, analysis tools or data; Wrote the paper.

## Data availability statement

Data will be made available on request.

## Declaration of competing interest

The authors declare that they have no known competing financial interests or personal relationships that could have appeared to influence the work reported in this paper.
